# Chemopreventive Effect of 5-Flurouracil Polymeric Hybrid PLGA-Lecithin Nanoparticles against Colon Dysplasia Model in Mice and Impact on p53 Apoptosis

**DOI:** 10.3390/biom11010109

**Published:** 2021-01-15

**Authors:** Mohammed A. Attia, Eman T. Enan, Abdullah A. Hashish, Sherif M. H. El-kannishy, Ahmed R. Gardouh, Mona K. Tawfik, Salwa Faisal, Amr El-Mistekawy, Ayman Salama, Suliman Y. Alomar, Amira H. Eltrawy, Sheka Yagub Aloyouni, Sawsan A. Zaitone

**Affiliations:** 1 Department of Clinical Pharmacology, Faculty of Medicine, Mansoura University, Mansoura 35516, Egypt; dr_moh_sas@mans.edu.eg; 2 Department of Pharmacology, College of Medicine, AlMaarefa University, Riyadh 11597, Saudi Arabia; 3 Department of Pathology, Faculty of Medicine, Mansoura University, Mansoura 35516, Egypt; emanenan@mans.edu.eg; 4 Department of Clinical Pathology, Faculty of Medicine, Suez Canal University, Ismailia 41522, Egypt; aahashish80@med.suez.edu.eg; 5 Basic Medical Sciences Department, College of Medicine, University of Bisha, Bisha 61922, Saudi Arabia; 6 Department of Toxicology, Mansoura Hospital, Faculty of Medicine, Mansoura University, Mansoura 35516, Egypt; Sherifhassan@mans.edu.eg; 7 Department of Pharmacology and Toxicology, Faculty of Pharmacy, University of Tabuk, Tabuk 71491, Saudi Arabia; 8 Department of Pharmaceutics and Industrial Pharmacy, Faculty of Pharmacy, Suez Canal University, Ismailia 41522, Egypt; ahmed_mahmoud@pharm.suez.edu.eg; 9 Department of Pharmacy, Faculty of Pharmacy, Jadara University, Irbid 21110, Jordan; 10 Department of Clinical Pharmacology, Faculty of Medicine, Suez Canal University, Ismailia 41522, Egypt; 11 Department of Biochemistry and Molecular Biology, Faculty of Medicine, Suez Canal University, Ismailia 41522, Egypt; dr_salwafaisal@med.suez.edu.eg; 12 Department of Internal Medicine, Gastroenterology Division, Faculty of Medicine, Al-Azhar University, Cairo 11651, Egypt; drmistekawy@azhar.edu.eg; 13 Department of Pharmaceutics, Faculty of Pharmacy, University of Tabuk, Tabuk 71491, Saudi Arabia; agrwan@ut.edu.sa; 14 Department of Pharmaceutics and Industrial Pharmacy, Faculty of Pharmacy (Boys), Al-Azhar University, Nasr City, Cairo 11751, Egypt; 15 Doping Research Chair, Department of Zoology, College of Science, King Saud University, Riyadh 11495, Saudi Arabia; 16 Department of Anatomy and Embryology, Faculty of Medicine, Alexandria University, Alexandria 22785, Egypt; dr.amiraeltrawy@gmail.com; 17 Health Sciences Research Center, Princess Nourah Bint Abdulrahman University, Riyadh 84428, Saudi Arabia; syaloyouni@pnu.edu.sa; 18 Department of Pharmacology and Toxicology, Faculty of Pharmacy, Suez Canal University, Ismailia 41522, Egypt

**Keywords:** apoptosis, 5-flurouracil, experimental colon dysplasia, mouse, polymeric lipid hybrid nanoparticles, PLGA-lecithin

## Abstract

The use of 5-fluorouracil (5FU) is associated with multifaceted challenges and poor pharmacokinetics. Poly(lactic-co-glycolic acid)-lipid hybrid nanoparticles (PLNs)-based therapy has received attention as efficient carriers for a diversity of drugs. This study evaluated the in vivo chemotherapeutic and anti-proliferative efficacy of 5FU-loaded PLNs against 1,2-dimethylhydrazine (Di-MH) prompted colon dysplasia in mice compared to free 5FU. 5FU PLNs were prepared. Male Swiss albino mice were distributed to six experimental groups. Group 1: Saline group. All the other groups were injected weekly with Di-MH [20 mg/kg, s.c.]. Group 2: Di-MH induced colon dysplasia control group. Groups 3 and 4: Di-MH + free 5FU treated group [2.5 and 5 mg/kg]. Groups 5 and 6: Di-MH + 5FU-PLNs treated group [2.5 and 5 mg/kg]. Free 5FU and 5FU-PLNs doses were administered orally, twice weekly. Treatment with 5FU-PLNs induced a higher cytoprotective effect compared to free 5FU as indicated by lower mucosal histopathologic score and reduction in number of Ki-67 immunpositive proliferating nuclei. Additionally, there was significant upregulation of p53 and caspase 3 genes in colon specimens. Our results support the validity of utilizing the PLNs technique to improve the chemopreventive action of 5FU in treating colon cancer.

## 1. Introduction

Colorectal cancer is the most common cancer affecting the digestive tract and is a main leading cause of death worldwide [1]. Characteristics of colorectal cancer involve uncontrolled cell proliferation and growth involving colonic crypt epithelial lining cells, beginning with hyperplasia and slowly evolving into invasive carcinoma [2]. Several animal models of colon cancer have been developed in order to explore its molecular pathogenesis and to investigate the role of various potential preventive nutritional and pharmacologic agents [3]. Among the various chemically prompted animal models of dysplastic colon, the 1,2-dimethylhydrazine (Di-MH) model is universally utilized [4,5].

Significant progress in chemotherapy for colorectal cancer has been made, in which 5-fluorouracil (5FU) is still representing one of the cornerstones and most active anti-cancer drugs among them. However, 5FU is far from perfect as its usage is associated with multifaceted challenges and dose-related toxicities due to the off-target accumulation and poor pharmacokinetics, which limits its therapeutic effectiveness and susceptibility to multi-drug resistance during treatments [6]. Nowadays, delivering safe and efficient doses of drugs is an ultimate goal of modern cancer chemotherapy; these doses will target the disease sites and spare the normal tissues [7]. Therefore, a selective drug delivery system to address the limitations of conventional therapies is required [8,9].

Nanodrug-delivery systems have gained great interest as an effective drug delivery system, thus representing an innovative approach for controlled release and targeted delivery of the drug to cancers [10,11] as they may minimize the toxic effect and enhance the anticancer treatment efficacy. Among the nanotechnology-based drug-delivery systems, liposomal systems and polymeric nanoparticles are acquiring interest as they have biodegradable properties that were approved for clinical use [12]. The recent progress in biomolecular therapeutics seeks to design new smart strategies for active targeting drug delivery approach, which would guarantee the optimum bioavailability of the encapsulated active pharmaceutical ingredient [10,11,12].

The poly(lactic-co-glycolic acid) (PLGA)-based materials, are gaining considerable attention and represent one of the most utilized polymers in formulating drug delivery systems as they overcome the limitations of lipids and polymers and combine the advantages of polymeric NPs as well as liposomes; offering enormous potential in the field of nanmedicine [9,13,14,15,16,17,18] by enhancing drug pharmacokinetics and distribution in the biological environment accompanied with a reduction of their side effects [9,19,20,21,22,23].

Polymer (PLGA)-lipid hybrid nanoparticles (PLNs)-based therapy has received attention as highly useful carriers for a wide range of dosage forms, especially in the preparation of chemotherapeutic medications. Lecithin was used in this study as a lipid material with PLGA to prepare PLNs as a new generation of polymer nanoparticles in which a lipid is required to coat the polymer core and stabilize formed particles. Furthermore, the PLNs prevent most of the drawbacks of polymer nanoparticles or lipid nanoparticles such as the burst release [24,25].

Our study used a Box–Behnken factorial design to choose runs depending on independent variables like surfactant, lipid, and polymer concentrations and we obtained 15 runs that were prepared according to the modified nanoprecipitation technique. The formula of lowest particle size and smallest polydispersity index (PDI) and highest encapsulation efficacy (EE%) was in a complete agreement with the predicted values from the experimental design; this encouraged us to carry out further in vivo screening of chemopreventive activity.

To date, no previous studies have formulated 5FU in PLNs or tested the antitumor activity in animal models of colon cancer. Therefore, this study was conducted to evaluate the in vivo chemopreventive efficacy of 5FU-loaded PLNs against Di-MH induced colon hyperplastic growth in mice and compared to free 5FU.

## 2. Materials and Methods

### 2.1. Drugs and Chemicals

5FU and poloxamer 188 (Pluronic F68) were purchased from Sigma-Aldrich Co. (St. louis, MO, USA). PLGA (RESOMER^®^ Condensate RG 50:50 Mn 2300) was gifted from Evonik, Essen, Germany, with M.Wt 2000–2500 g/mole. Lecithin was purchased from CISME Italy s.n.c. (Milan, Italy). All other chemicals and solvents were of HPLC grade and used as received without further purification.

### 2.2. Formulating the Hybrid Poly(Lactic-co-glycolic Acid) (PLGA)-Lecithin Nanoparticles

**Figure 1:**
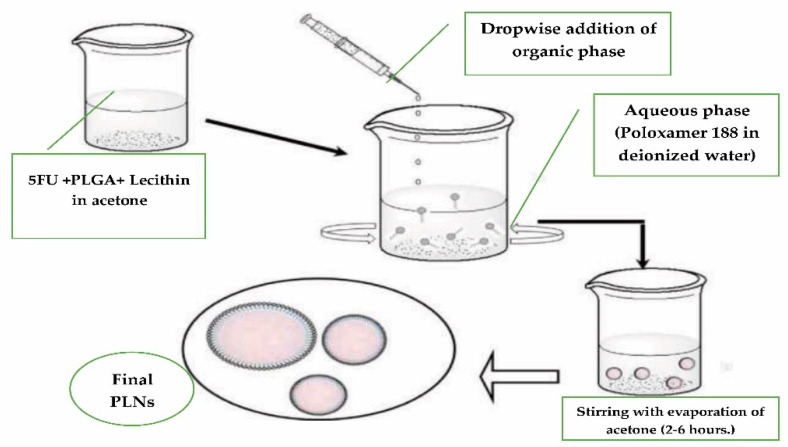
Schematic representation of the steps involved in mixed-polymer (PLGA)-lipid hybrid nanoparticles (PLNs) synthesis by modified single step nanoprecipitation method.

Nanoparticles of 5FU were formulated by a modified single step nanoprecipitation procedure [11]. Briefly, drug, polymer (PLGA), and lecithin were dissolved in an organic solvent (10% water-miscible solvent acetone), then added drop-wise into the surfactant (poloxamer 188) previously dissolved in deionized water (aqueous phase) by using a syringe (1 mL volume) with a fixed flow rate at 1 mL min^−1^ over a vortex. The formed solution was constantly stirred at 800 rpm with a magnetic stirrer (Remi, Mumbai, India) for 2–6 h (Figure 1).

### 2.3. Optimization of Formulation Variables

A statistical 3-factor and 3-level Box–Behnken design that yielded 15 runs was applied for performing the optimization study. Table 1 demonstrates the proposed independent and dependent variables of the formulated PLNs. A software-based assessment (Version 7 Design Expert^®^, Stat-Ease Inc., Minneapolis, MN, USA) was utilized to generate a polynomial equation based on quadratic model for formulating the preparations. The major components for the polymer (PLGA) lipid (lecithin) nanoparticulate system include the drug, polymer and lipid, and surfactant. These selections were based on their ability to produce small sized particles. The selections were also based on the safety profile and approval status of the components. Y_i_ = b_0_ + b_1 × 1_ + b_2 × 2_ + b_3 × 3_ + b_12_X_1_X_2_ + b_13_X_1_X_3_ + b_23_X_2_X_3_ + b_11_X^2^_12_ + b_22_X^2^_22_ + b_33_X^2^_32_, where, Y_i_ is the dependent variable; b_0_ is the intercept; and b from 1 to 33 are regression coefficients obtained from design. X_1_–X_3_ are independent variables that were chosen based on a pilot experiment.

The independent variables selected were concentration of PLGA (X1), concentration of lecithin (X2), and concentration of the surfactant (X3). The dependent variables were particle size (Y1) and entrapment efficiency (EE%) at high, medium, and low level (Y2). A checkpoint analysis was done to investigate the capability of the derived polynomial equation and contour plots to predict the responses [26]. To optimize the conditions, the level of independent variables (X1, X2, and X3) that would produce the minimal particle size (in nm) (Y1) and maximum value of EE% (Y2) was selected.

### 2.4. Characterization and Evaluation of the Optimized Formula

#### 2.4.1. Particle Size, Polydispersity Index, and Zeta Potential (ZP)

The average particle size (in nm), PDI and ZP (in mv) of the PLNs were evaluated by using a Mastersizer Hydro MU 2000S, Malvern MU Instruments (Malvern, UK), Malvern Zetasizer Nano-ZS90 (Malvern, UK), built-in dynamic light scattering (DLS) and laser doppler electrophoresis. The samples were put in ‘folded capillary cells’ and results obtained for size, PDI, and zeta potential were recorded. The control volume of the sample was determined automatically by Zetasizer software (version 7.13), which manages the instrument that is known as obscuration level. Volume of samples for zeta potential in the cuvette was 1 mL while the angle of measurement was 13°.

#### 2.4.2. Encapsulation Efficiency (EE%)

Ultrafiltration centrifugation technique was used for the determination of the encapsulation efficiency. One mL of the nanoparticle suspension was centrifuged at 12,298 RCF (Hettich centrifuge, Mikro 22R, Tuttingen, Germany) for 10 min. The solution containing the free drug was taken from the bottom of an Amicon tube and estimated by spectrophotometric analysis (266 nm, GENESYS 10 S UV-VIS Spectrophotometer, Thermo Scientific, Fisher Scientific-Arendalsvagen, Goteborg, Sweden). Calculation of the drug encapsulation inside nanoparticles was done by dividing the alteration between the total amount used (W_total_ 5FU) and the free amount presented in the supernatant aqueous phase (W_free_ 5FU) by the total amount used of 5FU, according to the following formulae:

(Encapsulation efficacy %) = (W_total_ − W_free_)/(W_total_) × 100 (1)

W_total_ = drug weight in the formulation;

W_free_ = drug weight in the formulation.

#### 2.4.3. In Vitro Drug Release of Mixed 5FU-PLNs

One mL of the nanoparticle suspension was moved to a dialysis tube (MWCO = 12,000 g/mole), tightened from both ends, and then added to 50 mL of phosphate buffered saline (PBS) dissolution medium (pH 6.8) at 37 °C in a shaking water bath. One mL of dialysis medium was removed at preset time intervals and replaced with fresh media. The drug release at different time points was estimated by reading the color intensity with a spectrophotometer at 266 nm (GENESYS 10S UV visible spectrophotometer, Thermo Scientific, Fisher Scientific-Arendalsvagen, Goteborg, Sweden).

The volume of dissolution medium was 50 mL (sink condition means that dissolution medium is at least 10 times the saturation solubility, for a sample containing 33.3 mg of the drug %). For the chosen pH, we found that release of the drug in acidic buffer (pH 1.2) for 2 h was less than 25%. Hence, we carried out the release study in pH = 6.8. In agreement with us, one study tested 5FU PLGA nanoparticles at pH = 7.4 not in an acidic pH [27].

### 2.5. In Vivo Pharmacological Activity

#### 2.5.1. Experimental Animals

Sixty male Swiss albino mice (body weight = 20–25 g) were provided by Hamada Abdelhaleem Company (Giza, Egypt) and acclimatized to the testing conditions. All the experimental procedures were accepted by the Research Ethics Committee (#201907RA2) at the Faculty of Pharmacy, Suez Canal University.

#### 2.5.2. Drugs and Chemicals

1,2-dimethylhydrazine (Di-MH) was supplied by Sigma-Aldrich (St. Louis, MO, USA) and diluted with sterile saline. Mice received weekly doses of Di-MH (20 mg/kg) by subcutaneous (s.c.) injections for 16 weeks [5,28] for induction of colonic dysplasia. Indeed, the main route of administration of Di-MH is the subcutaneous route [4,29]. Furthermore, intraperitoneal injections were also successful to produce colon tumors [30,31]. 5FU was given by oral gavage twice weekly from the beginning of week 9 until the end of week 16 at doses equal to 2.5 and 5 mg/kg [32].

#### 2.5.3. Experimental Groups

Mice were distributed to six experimental groups (10 mice in each group). Group 1: Saline group: normal mice injected with saline once weekly (s.c.) for 16 weeks. Group 2: Di-MH induced colon dysplasia control group with mice injected weekly with Di-MH and received twice weekly oral doses of the 5FU vehicle (distilled water) starting from the beginning of week 9 and continuing until the end of week 16. Groups 3 and 4: Di-MH + free 5FU treated group where mice received twice weekly doses of 5FU [2.5 and 5 mg/kg, by oral gavage]. Groups 5 and 6: Di-MH + 5FU-PLNs treated group where mice received twice weekly doses of 5FU-PLNs [2.5 and 5 mg/kg, by oral gavage].

#### 2.5.4. Animal Sacrifice and Blood/Tissue Sampling

Mice were anesthetized with ketamine and killed by cervical dislocation. Tissue specimens from the descending colons were removed, rinsed with ice-cold PBS and divided into two pieces. The first piece was frozen at −80 °C and was later homogenized and directed for the polymerase chain reaction (PCR) assays. The second piece of each colon specimen was dissected, fixed in 10% paraformaldehyde solution, and embedded in paraffin wax for subsequent use for histopathological routine staining and immunohistochemistry.

#### 2.5.5. Determination of Colonic Expression of p53 and Caspase 3

##### RNA Extraction

Homogenized colon specimens were lysed for isolation of the total RNA (t-RNA) using the Qiagen RNAeasy Kit (Germantown, MD, USA), which was used following the guidelines listed by the manufacturer. RNA quantity and purity were estimated by a Beckman dual spectrophotometer (USA). The utilized absorbance ratio was 260/280 nm. Furthermore, 1% agarose gel electrophoresis was used to estimate the integrity of the isolated RNA.

##### Real Time-PCR Detection of p53 and Caspase 3 Gene Expression

For quantitative assessment of caspase 3 and p53 gene expression, 10 ng of the t-RNA extracted from each sample were reverse transcribed to cDNA by the aid of the Applied Biosystems high capacity cDNA Reverse Transcriptase Kit (USA). Amplification of the cDNA was done in a 48-well plate by the SYBR Green I PCR Master Kit (Fermentas, Waltham, MA, USA); this reaction was done using the Step-One PCR instrument (Applied Biosystem, Foster City, CA, USA). The volume of each PCR reaction mix was 20 μL and prepared as follows: 10 μL SYBR Green I master mix, 7 μL PCR grade water, 1 μL of 10 ng cDNA, and 1 μL (1 μM) for each forward and reverse primer as illustrated in Table 2. The thermal cycle conditions were adjusted in a Step-One instrument as follows:10 min at 95 °C for enzyme activation followed by 40 cycles of 15 s at 95 °C, 20 s at 55 °C, and 30 s at 72 °C. The PCR amplicons were established by melting curve analysis. The relative expression of each of the target genes was normalized to a house keeping gene (glyceraldehyde 3-phosphate dehydrogenase (GAPDH). The results were calculated as fold change in gene expression by comparative Ct method (2^−ΔΔCt^) [33]. The Ct values were calculated by Step-One PCR software.

#### 2.5.6. Histopathological Examination of Colon Tissue

Paraffin embedded colon tissues were cut into 5 µm thick sections and mounted on glass slides. Following dewaxing and rehydration, one set of slides was stained with hematoxylin and eosin (H&E) and another set with Periodic acid Schiff (PAS). Slides were examined by light microscope for the evaluation of colonic mucosal lesions. Each section was given a score from 0 to 4 according to the presence and severity of hyperplasia or dysplasia. Foci in which the crypts were crowded elongated with serrated lumen and lined by a mixture of absorptive and goblet cells with crowded nuclei, but no stratification or atypia were considered hyperplastic lesions (score 1). Foci where the crypts showed loss of mucosal polarity and hyperchromasia of nuclei with nuclear stratification were classified as dysplastic. Dysplastic lesions were further graded into mild, moderate, and severe (score 2–4) [3]. PAS stained sections were assessed for the density of goblet cells and % area of positive staining was measured using VideoTest Morphology software (Saint-Petersburg, Russia).

#### 2.5.7. Immunohistochemistry

Colon tissue sections were deparaffinized in xylene and rehydrated, followed by boiling in citrate buffer for antigen retrieval. Rabbit polyclonal antibodies for Ki-67 (ABclonal, Catalog #A2094, Woburn, MA, USA) were used for immunohistochemistry. The tissue specimens were inspected by a light microscope for photography; then the count of Ki-67 positive nuclei was measured in ten sequential sections from each animal and was evaluated by VideoTest Morphology software (Saint-Petersburg, Russia).

#### 2.5.8. Digital Morphometric Study

Photography was done using the 40X objective by the aid of an Olympus^®^ digital camera fixed upon an Olympus^®^ microscope with a ½-X photo adaptor. The photographs were examined by VideoTest Morphology^®^ software (Saint-Petersburg, Russia) with a definite built-in routine for measurement of the area % and object counting. Two parameters were quantified: the % of the area showing PAS positive staining and the number of Ki-67 immunpositive nuclei.

#### 2.5.9. Statistical Analysis and Data Presentation

Readings were expressed as mean ± standard deviation of the mean (SDM). Box–Behnken designs are experimental designs for response surface methodology, devised by George E. P. Box and Donald Behnken in 1960, to achieve the following goals. Each factor or independent variable, was placed at one of three equally spaced values, usually coded as −1, 0, +1. Meanwhile, results related to the biology experiment were analyzed using the version19 of SPSS program (SPSS Inc., Chicago, IL, USA). For quantitative variables, the normality of distribution was tested with the Kolmogorov–Smirnov test and the difference between variables was analyzed first by multivariate ANOVA and then one-way analysis of variance (ANOVA) whereas, Kruskal–Wallis ANOVA was applied for analysis of ordinal values of the histopathology score. Post-hoc tests were applied for multiple comparisons. The differences were assigned as statistically significant when the *p* value was less than 0.05.

**Figure 2:**
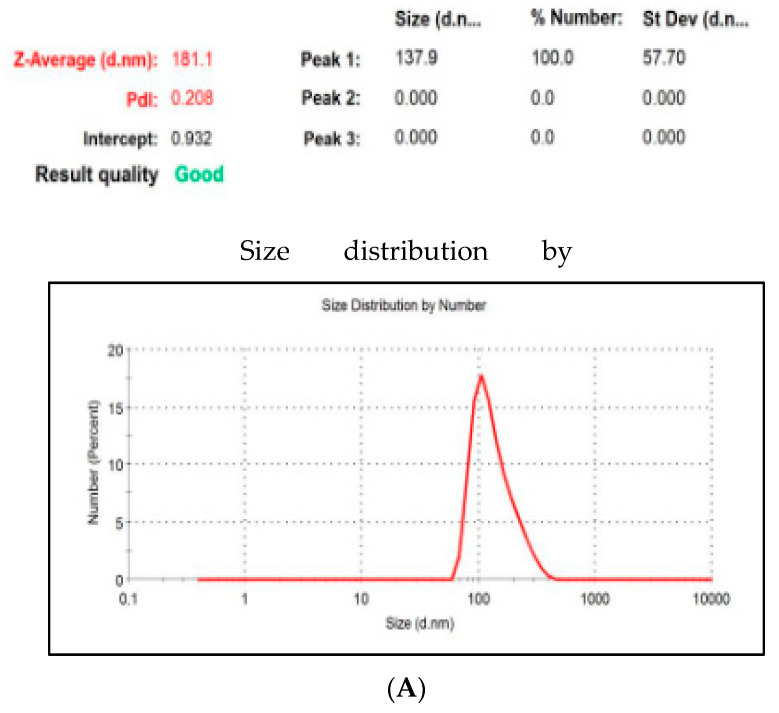

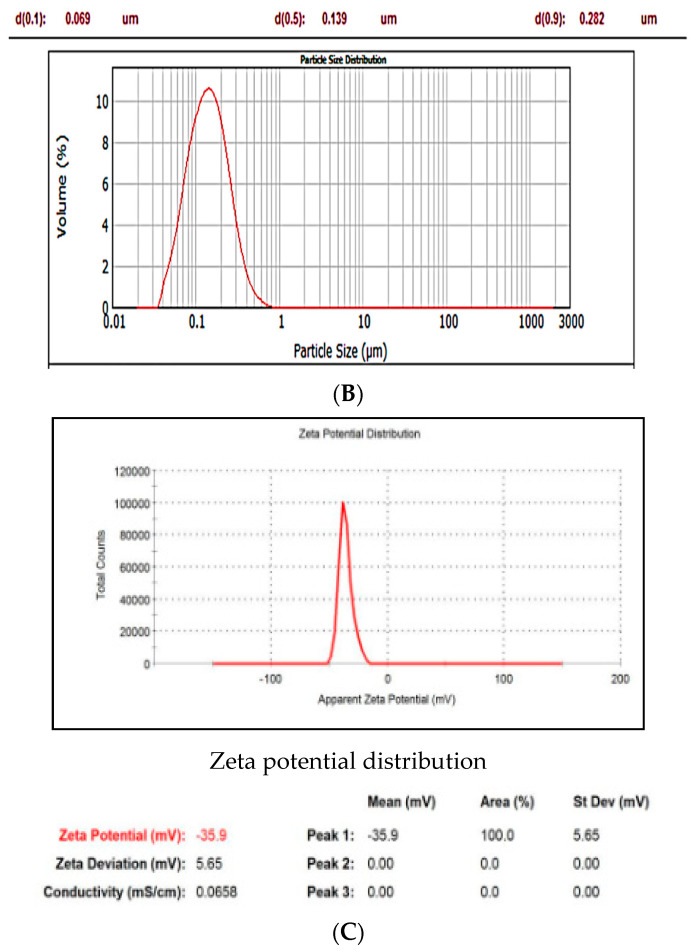
Particle size of the 5FU PLNs-11. Data show (**A**) particle distribution, (**B**) size distribution by volume, and (**C**) Zeta potential distribution. Figures demonstrate better polydispersity and narrow distribution, Z average size in nanometer range and zeta potential ensuring stability.

Average particle size of the formulae was determined at ambient temperature by dynamic laser light scattering apparatus. The Zeta potential showed that the developed formulations were stable and uniformly dispersed (Figure 2).

## 3. Results

### 3.1. Formulae Optimization and Characterizations

Fifteen experimental runs of PLNs prepared by the polymer, lipid, and surfactant were analyzed for particle size and put in the response column in the experimental design and presented in Table 3. A correlation between the different factors and formulation was established using the quadratic polynomial generated using the Box–Behnken design using the Design Expert^®^7.0.0 program. The particles’ sizes ranged from 138 nm to 210 nm. The entrapment efficiency (EE %) of PLNs was in the range of 58.7% to 75.6%. Overall, the influences of different independent variables on the PLNs were similar.

After determining the independent variables and their influence on the responses, optimum responses were selected. Hence, the optimal formula was assigned as the one that showed smaller particle size along with a high concentration of the entrapped drug. The variables related to the optimal formula are presented in Table 4.

**Figure 3:**
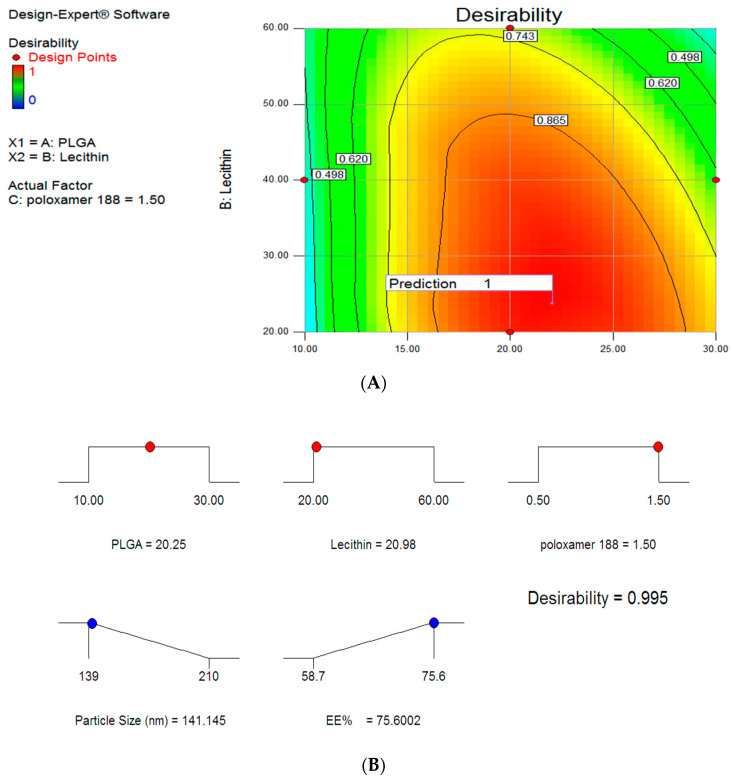
Desirability ramp for optimized conditions for PLNs process conditions. (**A**) represents the relationship between independent variables and desirability values, (**B**) obtained values from software to represents optimum conditions for PLNs, red bullets are of independent variables and blue ones are of dependent variables that give better desirability.

**Figure 4:**
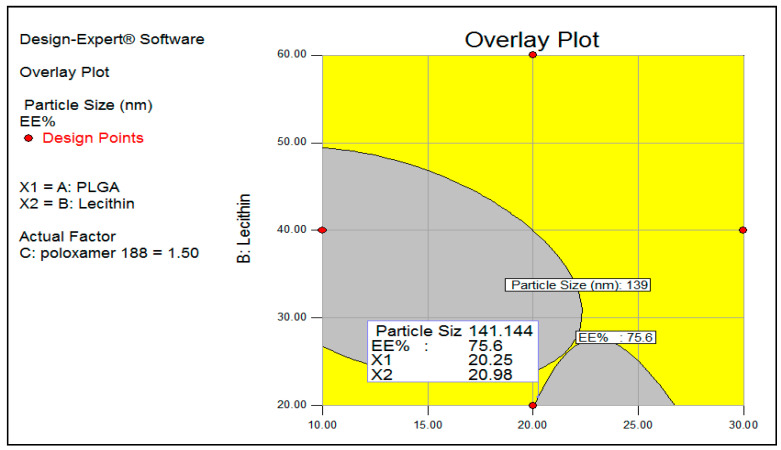
Design space for PLGA-lecithin 5FU nanoparticles. Gray areas show the possible situations to obtain the desired readings for a 141 nm average particle size and 75.6 EE%.

**Figure 5:**
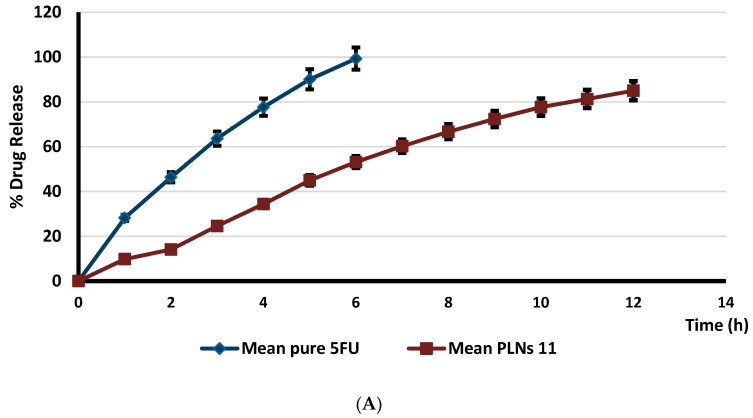

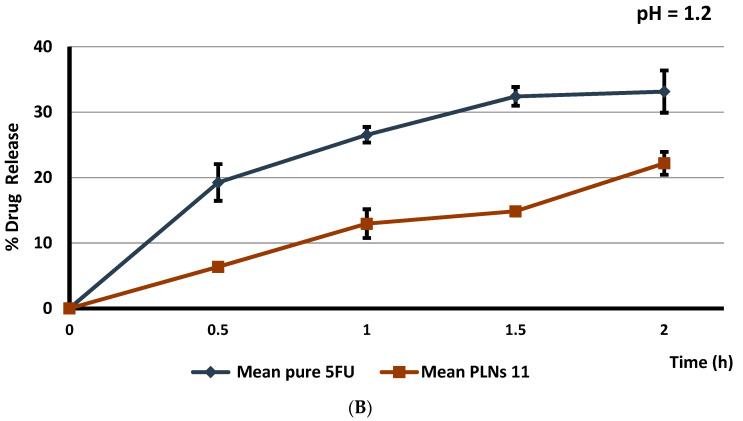
Release profile of 5-fluorouracil loaded on PLNs-11 at pH 6.8 (panel **A**) and pH 1.2 (panel **B**). Data are mean ± standard deviation for three replicates.

Figure 3 demonstrates the desirability of the optimized formula (0.948). The overlay plot describes the design space showing the optimum amount of the three independent variables (X1–X3) with required features of mean particle size (140 nm) and EE% (75.6%) (Figure 4). The in vitro release profile was investigated for the optimized formulae with anticipated particle size, Zeta potential, and entrapment efficiency. Mixed formulation for PLNs-11 and pure 5FU were studied using cellulose membrane as a semi-permeable membrane. Mixed PLNs formulation PLNs-11 showed not more than 25% release of 5FU at pH 1.2 (for 2 h), while at pH 6.8, the drug release was 53.13% (for 6 h). Pure drug released were 97% at pH 6.8 (for 6 h) (Figure 5). The cumulative percent of drug release for the PLNs-11 formula was found to be 90% at the end of about 12 h by sustainable drug released.

### 3.2. Results of the In Vivo Study

#### 3.2.1. Colonic Expression of the Target Genes

**Figure 6:**
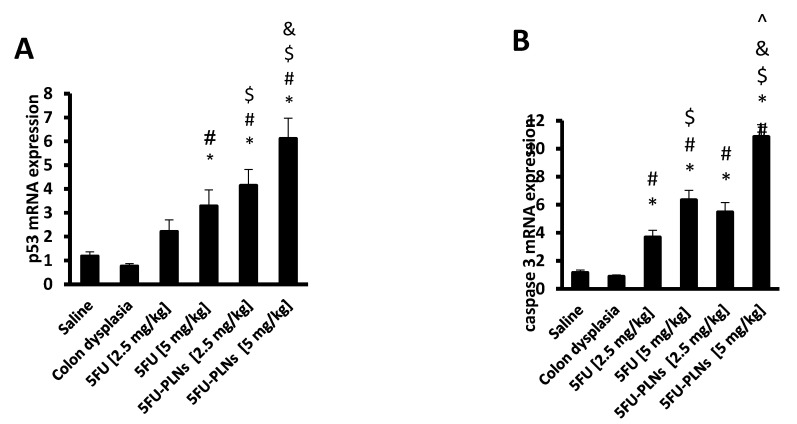
mRNA expression of p53 and caspase 3 genes in colon specimens. mRNA expression of p53 (**A**) and caspase 3 (**B**). Data are mean ± SDM and compared at p less than 0.05. *: Versus saline group; #: Versus colon dysplasia group; $: Versus 5FU [2.5 mg/kg] group; &: Versus 5FU [5 mg/kg] group; ^: Versus 5FU-PLNs [2.5 mg/kg] group.

mRNA expression of p53 and caspase 3 in colonic lysates indicated non-significant decreases in the colon dysplasia control group compared with the saline group (Figure 6A,B). Groups received free 5FU [5 mg/kg] and 5FU-PLNs [2.5 and 5 mg/kg] showed significantly upregulated expression of p53. The effect produced by the high dose of 5FU-PLNs was greater than that produced by the equivalent dose of free 5FU (Figure 6A).

In addition, free 5FU produced a dose-dependent rise in caspase 3 mRNA expression (Figure 6B). A similar dose-dependent effect was observed in mice treated with 5FU-PLNs (Figure 6B). Importantly, the effect produced by the high dose of 5FU-PLNs [5 mg/kg] was greater than that resulting from treating the mice with the free form of 5FU (Figure 6B).

#### 3.2.2. Histopathological Examination and Histologic Grading of Colonic Mucosal Lesions

**Figure 7:**
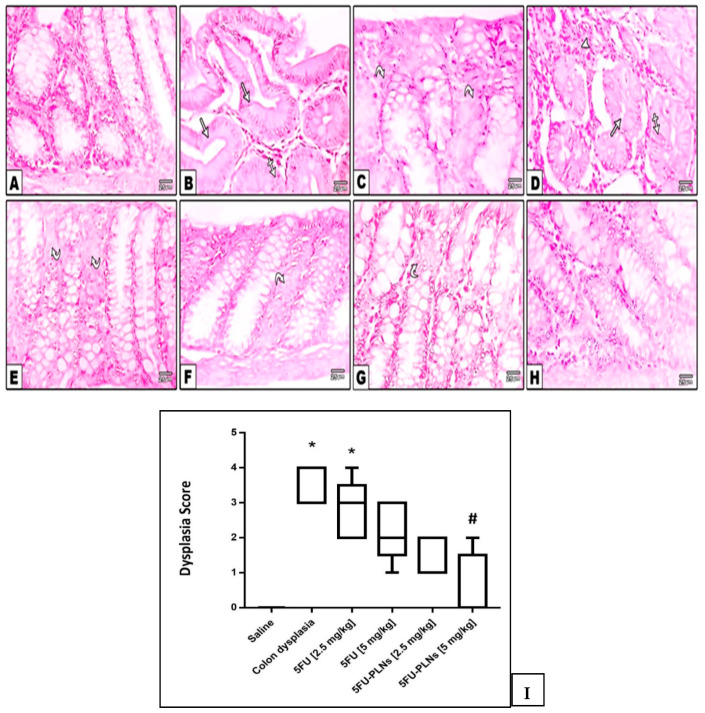
Photomicrographs of hematoxylin and eosin stained colonic specimens demonstrating intact crypts lined by normal appearing epithelial cells and goblet cells in the vehicle control group (**A**). In the colon hyperplasia control group, the foci of variable grades of hyperplasia and dysplasia were detected where crypts were enlarged, elongated, crowded, and irregular (straight arrows) lined by atypical closely packed epithelial cells having stratified hyperchromatic nuclei (crossed arrows), markedly reduced goblet cells accompanied with multifocal areas of cell proliferation (curved arrows), and dense inflammatory cell infiltration in lamina propria (arrow head) (**B**–**D**). In Free 5FU [2.5 & 5 mg/kg] treated groups, the crypts were less crowded, showing focal hyperplastic lining with small areas of cell proliferation (curved arrows) (**E**,**F**). In 5FU-PLNs [2.5 mg/kg] treated group, crypts were relatively straight, less crowded, with very small area of cell proliferation (curved arrow) (**G**). In the 5FU-PLNs [5 mg/kg] treated group, crypts were straight, lined by normal appearing epithelial cells and goblet cells (**H**) X400. (**I**) A box plot chart for the median histologic score and quartiles. Data were analyzed using the Kruskal–Wallis test and Dunn’s test at *p* < 0.05. *: Versus saline group, #: Versus colon dysplasia group.

On examination of the colonic specimens from different groups, no grossly remarkable lesions were observed. Microscopic inspection for H&E stained colon specimens revealed normal histologic structure of colonic mucosa, submucosa, and serosa (straight crypts lined with abundant mucus secreting goblet cells and columnar absorptive cells. Lamina propria contains scattered lymphocytes and plasma cells) in the saline control group, whereas colonic mucosa from the Di-MH control group showed foci of aberrant closely packed, elongated, and irregular crypts. The crypts were lined by hyperplastic to markedly dysplastic epithelium (large and hyperchromatic nuclei, loss of polarity, and cellular stratification) with diminished goblet cells and multiple foci of disorganized cell proliferation. The lamina propria showed moderate to severe chronic inflammatory cell infiltrate. Sections prepared from colon tissue in mice received 2.5 mg/kg of free 5FU. Interestingly, colonic tissues obtained from Di-MH + 5FU-PLNs treated mice revealed scarce to unremarkable histological changes. The crypts were more or less straight and regular, lined by normal appearing absorptive cells and vacuolated goblet cells. Only a few foci of cell proliferation were observed in the low dose group (Figure 7A–H). Panel I demonstrates the median score assigned to each experimental group; it is shown that the score given to colon dysplasia group and 2.5 mg/kg of free 5FU was greater than the saline group score, whereas the high dose free 5FU (5 mg/kg) and the low dose of 5FU-PLNs (2.5 mg/kg) caused a non-significant decrease in histologic picture compared with both colon dysplasia control groups (Figure 7I).

**Figure 8:**
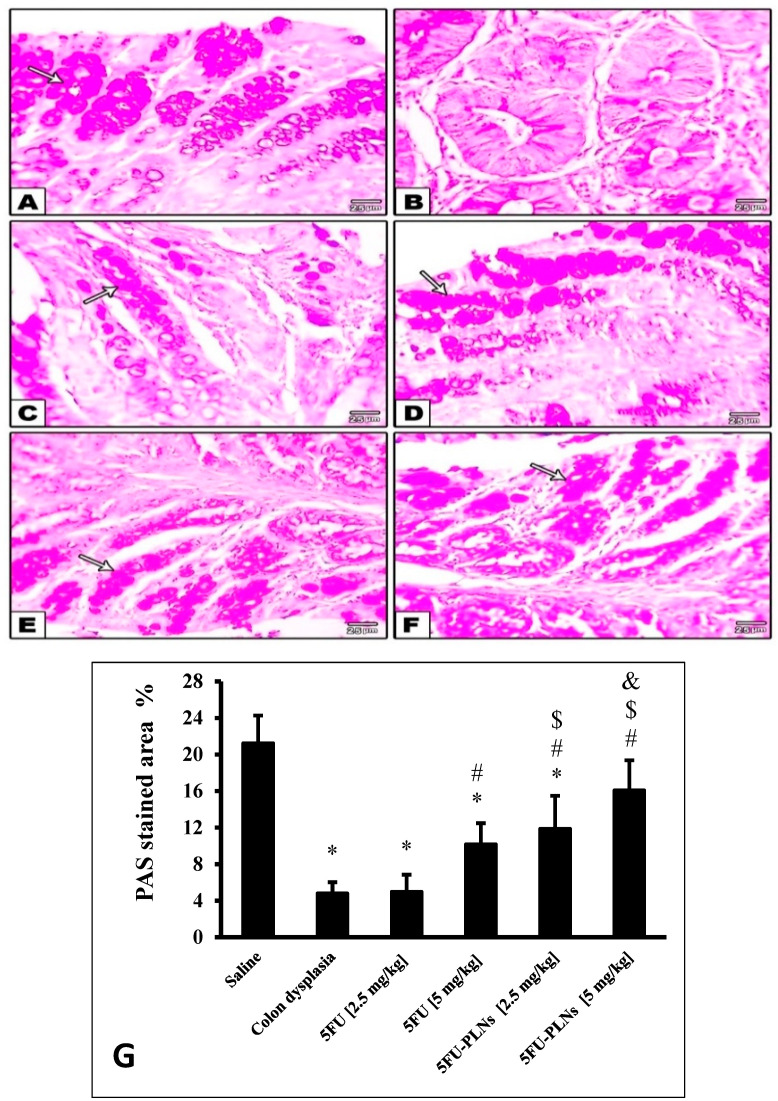
Microscopic pictures of periodic-acid Schiff stained colonic sections. Images show normal density of goblet cells (arrows) lining the crypts in the saline control group (**A**). Colon hyperplasia control group showed marked decrease of goblet cells in dysplastic crypts (**B**). Mice groups treated with free 5FU [2.5 or 5 mg/kg] (**C**,**D**) showed increased goblet cell density. Density of goblet cells appeared higher in groups treated with 5FU-PLNs [2.5 mg/kg] (**E**) and 5FU-PLNs [5 mg/kg] (**F**) X400. (**G**) Area of periodic acid Schiff staining presented as mean ± SDM and compared at p less than 0.05. *: Versus saline group; #: Versus colon dysplasia group; $: Versus 5FU [2.5 mg/kg] group; &: Versus 5FU [5 mg/kg] group.

Figure 8 demonstrates the photomicrographs for PAS stained colonic tissue. The saline group showed intact goblet cell population and preserved mucin content (Figure 8A). Image analysis and statistical analysis results indicate that the colon dysplasia group exhibited the most marked reduction of goblet cell area (Figure 8B). Treatment with low dose [2.5 mg/kg] free 5FU did not show any significant preservation of mucin content (Figure 8C), whereas, the high dose [5 mg/kg] free 5FU treated groups (Figure 8D) as well as both low and high doses 5FU-PLNs-treated ones (Figure 8E,F) revealed improved goblet cell population compared to the colon dysplasia group and 2.5 mg/kg of free 5FU treated group. Panel G demonstrates the area% of PAS staining and indicates noticeable decline in PAS staining in the colon dysplasia group and 2.5 mg/kg of the free 5FU group. Treatment with the 5 mg/kg of free 5FU or both doses of 5FU-PLNs produced significant elevations in PAS staining area; interestingly the PAS stained area in mice treated with 5 mg/kg of 5FU-PLNs was greater than that noticed in the mice group treated with an equal dose of free 5FU (Figure 8G).

#### 3.2.3. Immunohistochemical Expression of Ki-67 in Colon Tissue

**Figure 9:**
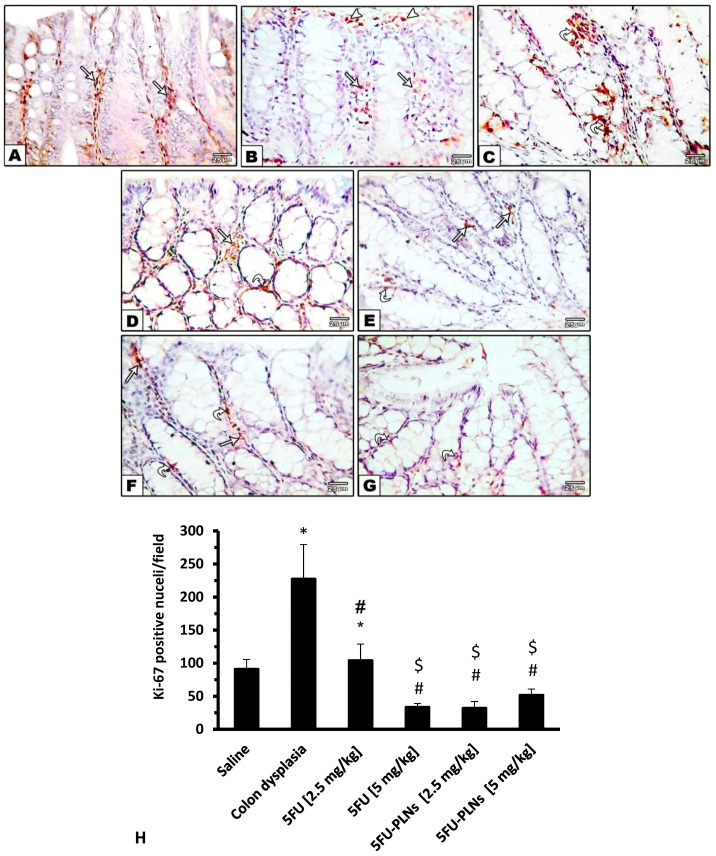
Microscopic pictures of immunohistochemical staining against Ki-67 of colon sections. Images for saline group (**A**), Colon dysplasia control group (**B**,**C**), FU [2.5 and 5 mg/kg] treated group (**D**,**E**), 5FU-PLNs [2.5 and 5 mg/kg] treated groups (**F**,**G**). Positive expression appears as brown nuclear staining of surface epithelial cells (arrow heads), crypt lining cells (straight arrows), and proliferating interstitial cells (curved arrows). Immunohistochemistry counterstained with Mayer’s hematoxylin X400. (**H**) Mean number of Ki-67 positive nuclei in experimental groups measured using VideoTest Morphology^®^ software (Saint-Petersburg, Russia) and presented as mean ± SDM and compared at *p* less than 0.05. *: Versus saline group; #: Versus colon hyperplasia control group; $: Versus 5FU [2.5 mg/kg] group.

Microscopic examination of the Ki 67-immunostained colon sections revealed a low number of stained nuclei in the saline group (Figure 9A), but a greater number was observed in the colon dysplasia group (Figure 9B,C). Mice treated with 2.5 mg/kg of free 5FU showed a low number of stained nuclei (Figure 9D) while the other treated groups showed very minor staining (Figure 9E–G). Statistical analysis of the readings obtained from image analysis highlighted a non-significant decrease in Ki-67 expression in mice treated with free 5FU (2.5 mg/kg) versus the colon dysplasia group, whereas the other treatment groups showed significantly lower number of Ki-67 stained nuclei (Figure 9H).

## 4. Discussion

Colon cancer develops through a slowly progressive, multistep process of mucosal epithelial cell dysregulation and abnormal proliferation [34]. The earliest findings in colon carcinogenesis models are characterized by diverse microscopically recognizable mucosal intraepithelial lesions. Histologically, these lesions display variable features ranging from mild hyperplasia to severe dysplasia [35,36]. Indeed, the Di-MH model is one of the most frequently used models of experimental colon carcinogenesis [3,37]. It induces many morphological and molecular alterations similar to those observed in human sporadic colon cancer including resemblances in the response to some chemopreventive agents [2,38].

In the current study, administration of the chemical carcinogen Di-MH induced substantial colonic mucosal hyperplastic and dysplastic changes of various grades. Mucosal crypts at dysplastic foci showed marked mucin depletion, and high expression of the cell proliferation marker Ki-67 in crypt lining cells compared to the vehicle control.

In cancer treatment, limited drug bioavailability, development of drug resistance, and toxic effect on different organs are major obstacles to successful chemotherapy. Nanotechnology has been widely used as a promising drug delivery tool that improves the bioavailability of cancer chemotherapy, intensifying its therapeutic efficacy with reduced drug resistance and side effects [39]. In the current study, we prepared 5FU-PLNs and investigated its cytoprotective effect against Di-MH induced colon dysplasia in mice compared to free 5FU.

Our results showed a significant improvement of colonic mucosal histology in sections obtained from mice treated with 5FU-PLNs compared to groups treated with free 5FU. The administration of both doses of 5FU-PLNs significantly decreased the mucosal epithelial dysregulation and improved the histological score with preservation of crypt uniformity and absence of epithelial hyperplastic or dysplastic changes. PAS staining also revealed increased goblet cell population. Additionally, significant reduction of cell proliferation was indicated by a diminished number of Ki-67 immuno-positive nuclei. The histopathological changes were mostly attributed to higher chemopreventive efficacy for the PLNs of 5FU.

Superior chemopreventive effect for the 5FU PLNs compared to the free 5FU was mostly attributed to improved drug targeting (delivery) by PLNs that mediated the sustained release and promoted preferential accumulation of 5FU in the tumor sites. Therefore, the antitumor effects of 5FU could be improved including proliferation suppression and apoptosis [40]. An in vitro study showed there was a burst release of 17.22% of FU at 7 h and furthermore, there was a controlled release up to 78.23% for 24 h. The nanoparticles exhibited a biphasic drug release pattern with initial accelerated release followed by sustained release over seven days [41].

Similarly different nano-formulations of 5FU were successful in enhancing their efficacy. For example, 5FU formulated in nanoparticles significantly exhibited a growth inhibitory effect on the examined colon cancer cell line [42]. Moreover, in vitro cytotoxicity studies using 5FU loaded polycaprolactone nanoparticles revealed greater anti-proliferative influence than free 5FU on the SW480 colon cancer cell line resisting chemotherapy [43]. One study used a molecularly imprinted nanoform of 5FU and tested its antitumor activity against Ehrlich solid tumor in mice and documented greater chemopreventive effect compared to the free form of 5FU [32]. Consequently, the authors concluded that 5FU loaded nanopreparations is a promising delivery system to target colon cancer [42].

Several studies have used the PLNs technology for formulating chemotherapeutic agents with positive outcomes. One study evaluated the in vivo efficacy of the doxorubicin-loaded PLNs formula was measured in a murine model of solid tumor. Assessment was dependent on measuring the delay in tumor growth in addition to histology and morphology of tumor and loco-regional distribution [44]. Another study used doxorubicin in a PLNs system; the authors documented increased uptake and retention in two different multidrug resistant cell lines compared to the solutions containing free doxorubicin [43].

One research paper utilized multiple layer-by-layer PLNs to improve the FOLFIRINOX combination, consisting of three chemotherapeutic drugs (5FU, irinotecan or oxaliplatin) and folic acid, which was efficient in treating pancreatic cancer patients with minimum side effects compared with the FOLFIRINOX regimen alone. Moreover, these PLNs showed higher efficacy than gemcitabine, which is the reference standard medication in this type of cancer [45].

Furthermore, another research group highlighted that the PLNs formula of doxorubicin enhanced cytotoxicity against breast cancer cells that were characterized by multidrug-resistance [46]. Another research group synthetized cRGD-directed, NIR-responsive and robust AuNR/PEG–PCL hybrid NPs for developing preparations containing paclitaxel or doxorubicin tested for efficacy against xenografts of human glioblastoma U87MG cells in nude mice. The in vivo pharmacokinetics studies showed that the NPs of doxorubicin showed longer circulation time, less adverse effects, and lower mortality if compared to the free form of doxorubicin [47].

In the present study, the colon cancer group showed a non-significant decrease in p53 and caspase 3 gene expression levels than the saline group. This finding might be related to the pattern of chemical induction of the colon dysplasia and gene expression was assessed at the dysplastic stage before establishment of a solid tumor. In agreement with our results, it was demonstrated that alterations of p53 gene expression in colon carcinogenesis occurred only at the late stages in the animal model [48].

In the current study, the p53 and caspase 3 genes were significantly overexpressed in the 5FU and 5FU-PLNs treated groups and the level of expression was proportional to the drug dosage. The gene overexpression appeared to be induced by 5FU as it might be capable of activating p53 expression by some proposed mechanisms such as incorporation of its metabolites into RNA and DNA as well as inhibition of thymidilate synthase [49]. The elevated p53 levels induced the proapoptotic B-cell lymphoma-2 (bcl-2) family expression. This is known to stimulate the release of cytochrome c from the mitochondria that eventually results in caspase 3 activation. In addition, activated p53 upregulates the expression of some death receptors, subsequently activating caspase 3 and inducing apoptosis [50,51].

In accordance, Cheng et al. [52] demonstrated higher expression of both p53 and caspase 3 mRNAs in 5FU, chitosan magnetics/5FU, and galactosylated chitosan-5FU nanoparticle treated groups of hepatocellular carcinoma in an orthotropic mouse model. Upregulation of p53 was found also in the fibrosarcoma skin tissues treated by 5FU NPs and 5FU [53]. Additionally, Abdel Latif et al. [49] found that 5FU increased the expression of the p53 gene in N-methylnitrosourea-induced colon cancer in rats. Moreover, several in vitro studies demonstrated the effectiveness of 5FU co-treatments in increasing p53 and caspase 3 protein expressions and activity [54,55,56,57].

The release profile of pure drug was higher than that entrapped in PLNs. This is because of the presence of PLGA and drug encapsulated in nanoparticles had a better influence on controlled release for a longer period of time, and can be confirmed by different studies that illustrated that the formulation of 5FU into polymeric nanoparticles had a better release profile than free drug, which suffers from rapid clearance from the body. In the case of the free drug, higher doses at short intervals are required to compensate the quick elimination and to maintain a steady state concentration to attain pharmacological activity. In the case of nanoformulation, the release of the drug does not demonstrate early burst release designating a complete encapsulation into the carrier matrix and absence of any superficial amount of unbound free nanoparticles. This is followed by sustained release and agrees with several previous reports [32,52,58,59].

It was previously documented that chemotherapeutic agents prepared in nanoformulations had stronger antitumor activity in several models of cancer. Utilizing a nanodrug delivery system was invented for anti-cancer drug transportation to colon cancer cells, while lessening unintended distribution for the drug in normal tissues [60,61]. NPs may help protect chemotherapeutic medications from first-pass metabolism, gastric and intestinal enzyme degradation; this results in an increased amount of the available drug for local delivery to the colon [62]. The limitation of the current work is the lack of in vivo toxicity study to determine the relative safety of 5FU PLNs compared to free 5FU.

Therefore, the current results highlight an attractive platform for in vivo oral chemotherapy targeting colon cancers. More studies are warranted to test the current formula of 5FU in other murine models of colon cancer to determine the toxicity and the degree of suitability to be tested in human studies in colon cancer patients.

## Figures and Tables

**Table 1:** Box-Behnken design of the polymer (PLGA)-lipid hybrid nanoparticles (PLNs).

Independent Variable				Levels		
				Low Coded (−1)	Medium Coded (0)	High Coded (1)
Factor	Name	Units	Type	Low Actual	Medium Actual	High Actual
**X_1_**	PLGA conc.	mg in 100 mL	Numeric	33.34	66.67	100
**X_2_**	Lecithin conc.	mg in 100 mL	Numeric	66.67	133.34	200
**X_3_**	surfactant	% (*w*/*v*)	Numeric	0.50	1.00	1.50
Dependent variables, Y1 = Particles size (nm); Y2 = % Entrapment efficiency.						

**Table 2:** Primer sequence and Gene Bank accession number for each gene.

Target Gene	Primer Sequence: 5′-3′	Gene Bank Accession Number
*p53*	F: CTAGCTCCCATCACTTCATCCC R: AAATGCAGACAGGCTTTGCAG	NM_001127233.1
*Caspase 3*	F: GAGCTTGGAACGGTACGCTA R: CCGTACCAGAGCGAGATGAC	NM_001284409.1
*GAPDH*	F: AGAGAGGCCCAGCTACTCG R: GGCACTGCACAAGAAGATGC	NM_008084.3

**Table 3:** Box–Behnken design with measured responses.

Run Formulation		PLGA	Lecithin	Surfactant	Particles Size	Entrapment
Code		mg	mg	(%)	(nm)	(%)
		X1	X2	X3	Y1	Y2
1	PLNs-01	−1	−1	0	153 ± 0.50	59.1 ± 0.15
2	PLNs-02	1	−1	0	155 ± 1.00	71 ± 0.58
3	PLNs-03	−1	1	0	172 ± 0.76	61 ± 0.20
4	PLNs-04	1	1	0	210 ± 1.53	65.5 ± 0.75
5	PLNs-05	−1	0	−1	154 ± 1.00	58.7 ± 0.61
6	PLNs-06	1	0	−1	152 ± 0.58	67.5 ± 1.00
7	PLNs-07	−1	0	1	140 ± 1.00	60 ± 0.55
8	PLNs-08	1	0	1	159 ± 1.00	69.3 ± 1.15
9	PLNs-09	0	−1	−1	140 ± 0.58	74.2 ± 0.20
10	PLNs-10	0	1	−1	191 ± 1.00	72.1 ± 0.51
11	PLNs-11	0	−1	1	139 ± 0.58	75.6 ± 0.25
12	PLNs-12	0	1	1	163 ± 1.53	72 ± 0.61
13	PLNs-13	0	0	0	150 ± 0.58	73 ± 0.25
14	PLNs-14	0	0	0	151 ± 1.00	72.7 ± 0.20
15	PLNs-15	0	0	0	149 ± 0.58	73.4 ± 0.25

**Table 4:** Optimized formulation as per the Design Expert^®^7.0.0 software.

**Name**	**Goal**	**Lower Limit**	**Upper Limit**	**Lower Weight**	**Upper Weight**	**Importance**
PLGA	Within range	33.3	100	1	1	3
Lecithin	Within range	66.7	200	1	1	3
Poloxamer 188	Within range	0.5	1.5	1	1	3
Particle size (nm)	Minimize	139	210	1	1	1
EE%	Maximize	58.7	75.6	1	1	5
Solutions						
**Number**	**PLGA** **(mg in 100 mL)**	**Lecithin** **(mg in 100 mL)**	**Poloxamer 188** **(1% *w*/*v*)**	**Particle Size (nm)**	**EE %**	**Desirability**
Software result 1	72.1	86.8	1.5	139.089	75.6	1
Software result 2	*67.5*	*69.9*	*1.5*	*141.145*	*75.6002*	*1 (selected)*
Software result 3	70.9	78.6	1.4	141.817	75.6	1

Table 4 contains data obtained from the software Design Expert, last column represents how this factor is important for optimization; 1 is most important while 5 is of least importance. Results 1, 2, and 3 were the different probabilities created by the software, which when related to runs, they expressed PLNs-11 and Result 2 was selected to be the best.

## Data Availability

Data are aavailable from S.A.Z. and A.R.G. upon request.
